# A Submersible, Off-Axis Holographic Microscope for Detection of Microbial Motility and Morphology in Aqueous and Icy Environments

**DOI:** 10.1371/journal.pone.0147700

**Published:** 2016-01-26

**Authors:** Christian A. Lindensmith, Stephanie Rider, Manuel Bedrossian, J. Kent Wallace, Eugene Serabyn, G. Max Showalter, Jody W. Deming, Jay L. Nadeau

**Affiliations:** 1 Graduate Aerospace Laboratories (GALCIT), California Institute of Technology, Pasadena, California, 91125, United States of America; 2 Jet Propulsion Laboratory, California Institute of Technology, Pasadena, California, 91125, United States of America; 3 School of Oceanography, University of Washington, Seattle, Washington, 98195, United States of America; Laval University, CANADA

## Abstract

Sea ice is an analog environment for several of astrobiology’s near-term targets: Mars, Europa, Enceladus, and perhaps other Jovian or Saturnian moons. Microorganisms, both eukaryotic and prokaryotic, remain active within brine channels inside the ice, making it unnecessary to penetrate through to liquid water below in order to detect life. We have developed a submersible digital holographic microscope (DHM) that is capable of resolving individual bacterial cells, and demonstrated its utility for immediately imaging samples taken directly from sea ice at several locations near Nuuk, Greenland. In all samples, the appearance and motility of eukaryotes were conclusive signs of life. The appearance of prokaryotic cells alone was not sufficient to confirm life, but when prokaryotic motility occurred, it was rapid and conclusive. Warming the samples to above-freezing temperatures or supplementing with serine increased the number of motile cells and the speed of motility; supplementing with serine also stimulated chemotaxis. These results show that DHM is a useful technique for detection of active organisms in extreme environments, and that motility may be used as a biosignature in the liquid brines that persist in ice. These findings have important implications for the design of missions to icy environments and suggest ways in which DHM imaging may be integrated with chemical life-detection suites in order to create more conclusive life detection packages.

## Introduction and Background

### Life in ice

Over the next several decades, both NASA and the European Space Agency (ESA) are likely to prioritize targets within the Solar System that contain abundant liquid water and ice: Mars [1], Europa, Enceladus, Titan, and Ganymede [2, 3]. While the first orbiter missions to the outer planet moons may examine only astrobiological potential, future landers and perhaps flybys with sample capture may directly search for extant life, especially microbial life. Missions to Mars to search for life in the polar ice caps have been proposed for launch within the next decade [4]. In order to maximize the chances of conclusive results, it is important to identify both *where* and *how* extant microorganisms would best be detected at these target sites.

**Fig 1:**
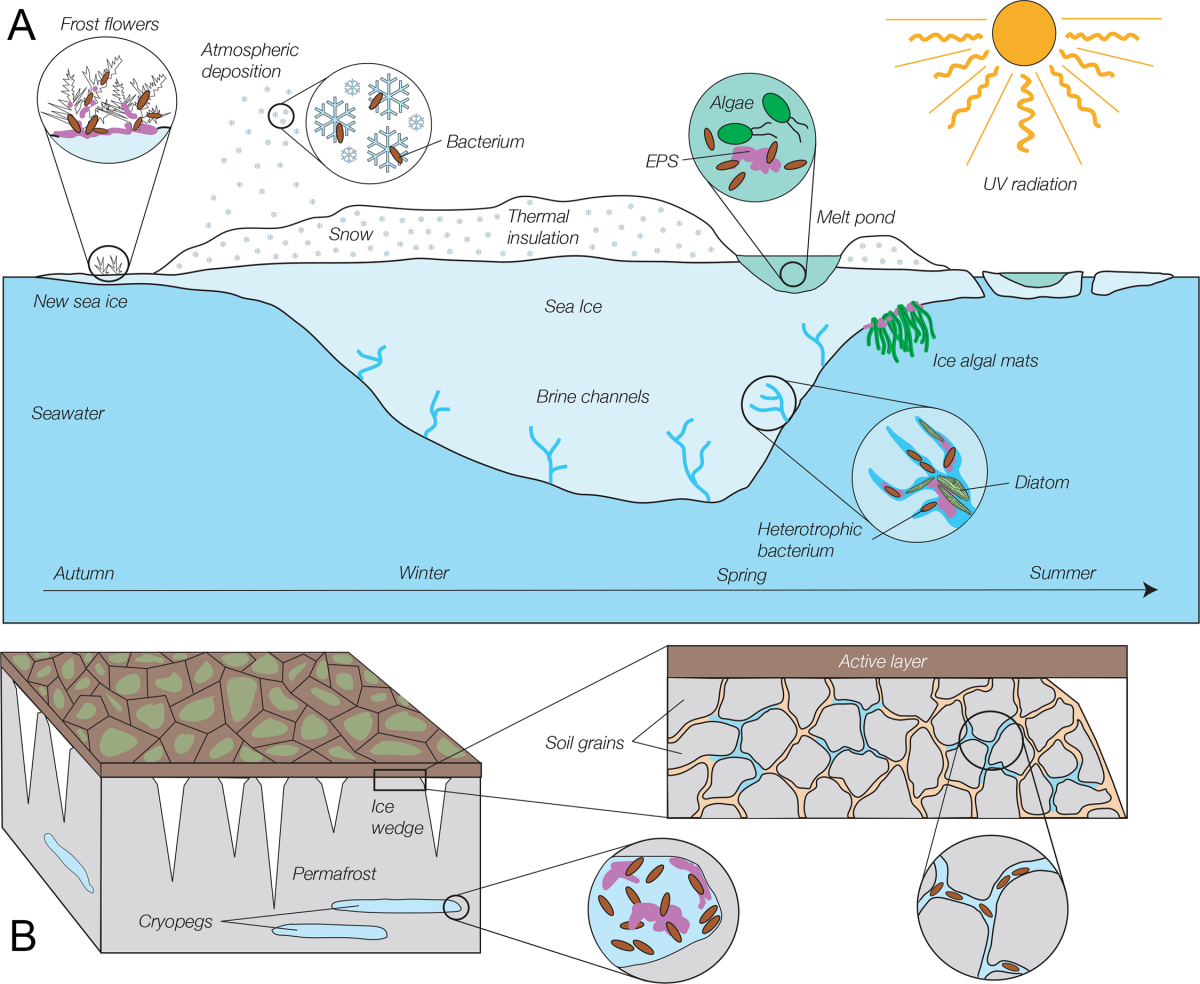
Schematic of selected microbial habitats in the frozen environments of sea ice and permafrost. (A) Sea ice (not drawn to scale). Microorganisms, in particular heterotrophic bacteria, inhabit all dimensions and seasons of sea ice and its snow cover, including thin first-year ice, ice structures on new ice called frost flowers, thick winter and spring ice, and surface melt ponds, despite exposure to high levels of potentially damaging radiation at the ice surface in summer. Most sea-ice bacteria derive from freezing seawater and inhabit the brine network within the ice, but bacteria delivered by atmospheric deposition are detected in overlying snow and surface melt ponds. Sea ice algae, especially diatoms, bloom in spring and summer in the brine channels of bottom ice, where they are bathed with seawater nutrients and receive sufficient sunlight; they have also been found in large aggregates at the bottom of melt ponds and as filamentous mats on the underside of the ice. The porous ice matrix and frost flowers are filled with extracellular polymeric substances (EPS), which are also involved in attachment of under-ice algal mats. (See [11] [18], and [19] for more detail). (B) Permafrost (not drawn to scale). In permanently frozen soil (grey), below the seasonally active layer (dark brown), bacteria and archaea can be found in abundance in cryopegs (buried lenses of relict seawater brines), where EPS concentrations are also high, and in veins of liquid brine that can exist between mineral grains. Freshwater ice wedges (white) that extend into permafrost also contain intact microorganisms, but at far lower abundances than in cryopegs or permafrost veins (See [13]and [14] for more detail).

Most discussions of astrobiology on the icy moons concern the ability of a lander to access the subglacial ocean, although realistic instrument descriptions for the 2020–2030 time frame anticipate being able to probe depths of at most a few meters, insufficient to reach the ocean on Europa [5, 6]. However, recent findings across Earth’s Polar regions suggest that it may be necessary to drill only deeply enough to access ice that has not been sterilized by ionizing radiation in a location free of meteor impacts in order to find microorganisms. Some of the most remarkable findings in astrobiology relate to the extreme conditions under which recoverable microorganisms may be found on Earth [7], including within all ice environments that have been studied, even those hundreds of thousands of years old. Winter sea ice is the most extreme of these ice environments that still contains a sufficient liquid phase to support active microorganisms [8], specifically bacteria and archaea observed at temperatures as low as –20°C [9, 10]. A rich diversity of bacteria and eukaryotes exists in warmer (still subzero) sea ice (**Fig 1A**), with over 60 genera of bacteria reported for sea ice [11].

Permafrost is also rich in viable microorganisms [12] [13] (**Fig 1B**), with methanogens alone found in concentrations up to 10^7^ g^–1^, resulting in measurable methane fluxes from the active layer of permafrost. Studies of Siberian permafrost have reported more than 30 bacterial genera. Cryopegs, which are relic lenses of ancient seawater isolated for hundreds of thousands of years from the surface, also contain viable prokaryotes and microbial eukaryotes: up to 10^7^ organisms mL^–1^ observed, and up to 10^6^ mL^–1^ culturable [14, 15]. These cryopegs have been suggested as models for astrobiology environments [16, 17].

**Fig 2:**
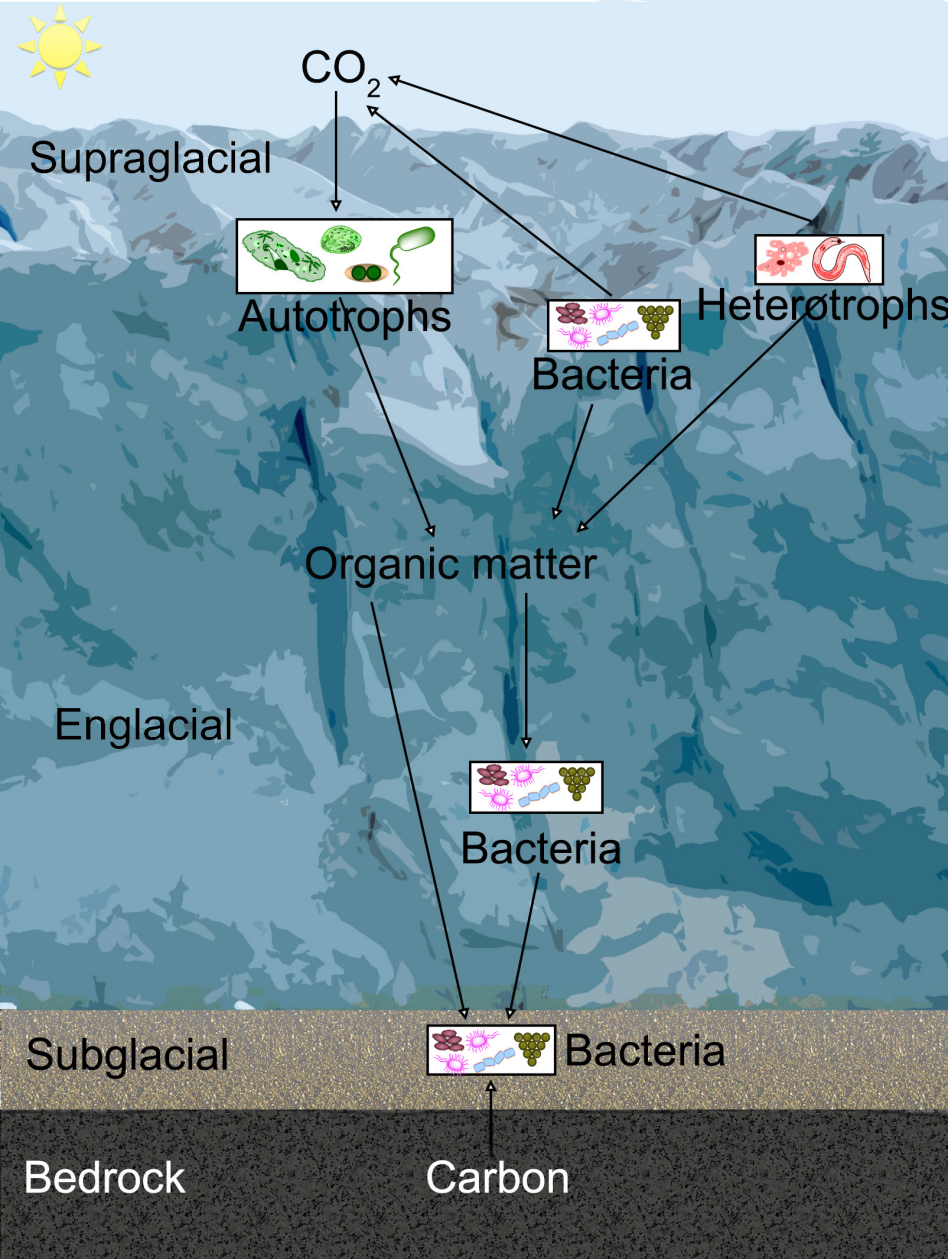
Glacial ecosystems showing simplified carbon cycle. Photosynthetic and heterotrophic prokaryotes and eukaryotes inhabit the supraglacial ecosystem, which receives sunlight and has abundant liquid water. Organic matter is slowly transferred to englacial organisms and more rapidly transferred via hydrological processes to subglacial bacteria. The subglacial surface may be till or water, as in the case of subglacial lakes.

Both prokaryotes and eukaryotes have also been found in glacier ice, including ice many thousands of years old in both the Northern and Southern hemispheres [20] [21] [22] [23] [24]. Glacier ice exhibits three distinct ecosystems: the supraglacial (surface) [25] [26] and subglacial (ice-bed interface) ecosystems, and finally that found directly within glacier ice (the englacial environment) (**Fig 2**). The englacial ecosystem has been less well studied because the overall biomass and metabolic activity are lower and so play a small role in the overall ecology of earth glaciers. However, active organisms are known to exist there [27] [28]. Because englacial ice is oligotrophic and not exposed to large flora and fauna (e.g. birds, pollen grains), it represents an excellent extraterrestrial analog.

Organisms from ice environments have been studied by a variety of methods, including DNA analysis, epifluorescence examination of fixed specimens, incubation with tracers, and culturing, but rarely by direct observation without any prior treatment [29] [30] [31]. Many of these organisms are known to be viable, with the caveat that pockets of liquid water must exist in the ice or frozen soil in order for the organisms to be metabolically active *in situ*. However, all Earth ice is polycrystalline and contains water-filled veins and films of liquid water around ice crystals [32] [33]. Active metabolism within these pockets in glacial ice has been seen to at least –5°C using metabolic tests [34], but when culture-based methods are used, it is not certain whether the viable organisms are actively metabolizing or dormant *in situ*. If they are dormant, the nutrients needed to restore active metabolism and the timescale for reactivation remain largely unknown. Methods for immediate microscopic investigation of organisms from Earth’s cryoenvironments will shed light on the ability of organisms to survive and be active at subzero temperatures, and provide useful analogs for what might be observed elsewhere in the Solar System.

### Microscopy for space missions

What the hand lens is to geology, the microscope is to microbiology; if extant microorganisms are to be found in the Solar System, microscopes capable of detecting bacteria and archaea must be developed for planetary missions. There are a variety of imaging technologies appropriate for space flight, including “Atomic force microscopy… interferometry, scanning near-field optical microscopy, and electron microscopy techniques” [35]. Each has its advantages and drawbacks. The major disadvantages of light microscopy are limited throughput, need for expert manipulation in real time, and diffraction-limited spatial resolution. Bacteria and archaea are at the limit of resolution and contain fewer intracellular features than eukaryotic cells, so non-motile bacteria can be difficult to distinguish from debris. However, actively motile cells are readily distinguished under video microscopy, with swimming up to 100 times faster than Brownian motion and independent of any observable currents. Eukaryotic cells may be identified by light imaging alone. In both cases, a microscope should be part of an instrument suite that can help to confirm whether chemical signatures arise from life or from abiotic chemistry. Direct imaging can establish the existence of cells or cell-like objects, with or without motion. Microspectroscopy, mass spectrometry, and other techniques may then be applied to the “cells” to confirm that key biosignature organics (lipids, amino acids, nucleic acids) are present.

Holographic (interferometric) microscopy is a variation on light microscopy that is still diffraction-limited, but with a number of advantages over ordinary light microscopy that make it ideal for use in planetary space missions [36–38]:

- It has a large depth of field, because the entire volume contributes to the recorded hologram, and digital refocusing of samples can typically be achieved on 50x (or greater) the classical depth of field, with particle tracking and reduced resolution imaging over an even larger depth;
- Focus is achieved in the post-capture reconstruction process, so mechanical refocusing is not necessary; there are no moving parts that can jam or malfunction;
- It can be operated without user input, as all of the 3D information is recovered *a posteriori* from the hologram;
- It can produce simultaneous amplitude/intensity and quantitative phase images of the same field. The intensity is equivalent to bright-field light microscopy; quantitative phase has no exact counterpart, but like phase contrast, may be used to image transparent samples without dyes or stains.

In this article we report the design and performance of a field holographic microscope, a unique instrument that shows greater spatial resolution than all previous designs, allowing for imaging of microorganisms down to 800 nm. We also report on the use of this instrument for imaging and tracking the motion of single-celled organisms extracted directly from sea ice near Nuuk, Greenland, in Spring 2015. Motile organisms were seen in all samples, for a 100% “life detection” rate. Non-motile eukaryotes could be resolved by imaging alone, while non-motile prokaryotes could be identified (though not unambiguously) by introducing flow into the sample chamber. *In situ* motility at subzero temperatures was observed. The fraction of motile organisms and the swimming speed could be increased by warming the samples to +4°C; an increase in motile fraction could be obtained by the addition of simple nutrients (serine, trehalose) or by incubation with complete culture medium. The results presented here suggest that holographic microscopy is a useful technique for *in situ* observations in Earth cryoenvironments and lead to discussion of necessary steps to qualify the instrument for space flight.

## Materials and Methods

### Instrument description

Our team has reported two off-axis holographic microscope designs that demonstrate improved spatial resolution over commercial instruments [39] [40]. The most recently reported design has a common set of optics through which both a reference beam and object beam pass, making the device extremely robust, as it cannot be misaligned. This latter design has been made field-worthy as described below.

#### Mechanical

The complete instrument is in a self-contained, glass-filled polyester box that measures 60 x 25 x 12 cm. The total mass of the instrument is just under 10 kg without batteries, which add approximately 0.5 kg for each 99 Wh, 11 V Li-Ion battery. The instrument is sufficiently rugged that it can travel without an external hard case; plugs are taped into the sample carrier port and the instrument can be hand-carried in a soft-sided bag. The instrument is designed for compliance with air travel standards for passenger carry-on luggage; the instrument can be hand carried to avoid the risk of lost or delayed airline baggage or difficulty in shipping to remote locations. It has slight (~ 1 kg) positive buoyancy to reduce the risk of loss if the tether is lost when used in aquatic environments. Communication with the instrument is via a waterproof external Ethernet port and a WiFi interface interchangeably. The housing has LED battery status indicators and writes the current battery state, as well as other diagnostics, to a file that can be monitored via a remote connection. Image acquisition can be triggered by the user via external buttons or remote access, or through internal scripts that acquire images based on time, temperature, or other triggers.

#### Optics

**Fig 3:**
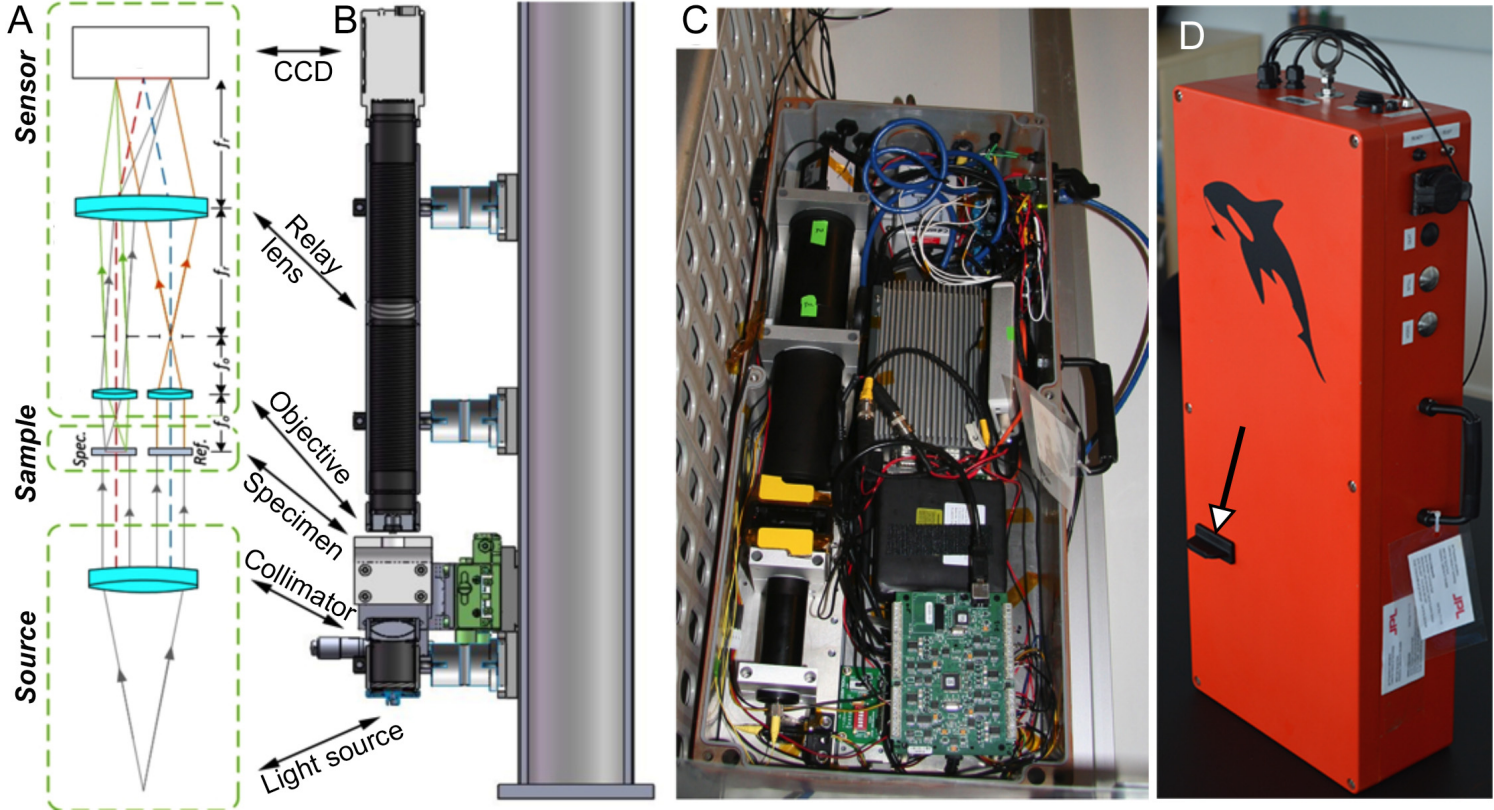
Schematic and images of the compact, twin-beam digital holographic microscope. (A) Schematic showing four main elements (discussed in the text): the source, the sample (specimen path is labeled *Spec*. and reference path is labeled *Ref*.), the microscope, and the sensor. (B) Solid model of the hardware. The fiber-fed source assembly is at the bottom, and the imaging camera is at the top. The microscope optics, comprised of the two aspheric lenses and the relay lens, are contained within the 300 mm long lens tube. In the laboratory, a three-axis stage between the source the microscope optics provides easy manual manipulation of the specimen under study. (C) Photograph of the field instrument (top case removed). The optical train, electronics, and computer are contained within a waterproof box. (D) Photo of instrument fully enclosed, as used in the field. The arrow indicates where a sample chamber is inserted; the structure pictured is a placeholder only.

A diagram of the optical system is shown in **Fig 3A**. This diagram captures the key components but is not to scale, so lengths and angles are not representative of the as-built system, as shown in the CAD drawing in **Fig 3B**. A photograph of the field instrument is given in **Fig 3C**. There are four main components to the system: the source, the sample, the microscope optics, and the sensor.

The source is a single-mode fiber-coupled laser diode, operating at 405 nm wavelength to optimize lateral resolution while avoiding phototoxic ultraviolet, and a one-inch diameter collimating lens. Optical power is tunable up to 10 mW to optimize for the camera shutter speed. The camera operates at shutter speeds from 25 microseconds to 43 s. The beam illuminates two volumes (sample and reference) that are spatially located ± 3 mm away from the optical axis. The use of the single mode input as a source ensures a high degree of spatial coherence. The microscope assembly is composed of a pair of aspheric singlet objectives followed by a common relay lens. Aspheric lenses have sufficient quality to yield diffraction-limited imaging capabilities but avoid the cost and complexity of compound objectives.

The final component in the system is the imaging sensor (Baumer TXG-50 or AVT Prosilica GT2450B, both using a Sony ICX-625 CCD sensor), selected for large field of view, small pixels, and rapid readout. The main requirement for the camera is that the fringes be oversampled to accommodate the bandwidth of the magnified sample while separating the interference terms in the Fourier domain with no overlap [9]. The optical elements are mounted in a commercially available lens tube assembly, allowing for a mechanically stiff, low cost system. The asphere holder is an inexpensively 3D-printed part.

#### Electronics and software

The on-board electronics are based around a low-cost, rugged COTS computer (Logic Supply POC-210) designed for automotive applications with components selected for a −30 to +70°C operating temperature range. The computer is equipped with two Gigabit Ethernet ports and 5 USB ports, as well as 802.11b g^–1^ wireless, and digital I/O. It typically uses less than 8 W and is rated at < 13 W with CPU, GPU, GigE at 100% with a 2.5-inch hard drive. The instrument normally runs without a display and with a solid state drive, reducing the required power below this tested rating. A 500 GB external solid state drive inside the instrument box is used for image and data storage. The operating system is Ubuntu Linux, which is well supported by data acquisition hardware to simplify development and also allows the user to limit background tasks that consume system power or interrupt data acquisition.

Additional electronics for data acquisition and control are an 8 channel, 12-bit A/D, D/A, digital I/O card (Measurement Computing USB1208FS-plus-OEM), an 8 channel temperature card for that can read thermocouples and resistive temperature sensors (Measurement Computing USB-TEMP), a power-over-ethernet (PoE) box, and a mechanical relay board for switching power. The PoE box supplies power to the camera and can be switched on and off via the relay card to turn the camera on and off (the camera uses about 1/3 of the instrument power) for extended operating time. The relay board is also used to control the pump and valves of the microfluidics.

The A/D board is used to monitor and control the laser power, read button inputs, and drive LED indicators on the instrument case. This board also monitors internal moisture sensors that alert the user if water is leaking into the electronics space of the instrument. The temperature card is connected to sensors that monitor the sample temperature, laser diode temperature, camera temperature, computer temperature, and external temperatures. Sample temperature can be monitored with very small sensors built into the sample chambers or larger sensors built into the input/output lines connected to the sample chamber.

The laser power is controlled by a D/A output that is limited by a resistor network to keep the laser below 85% of its maximum rated power to prevent damage, even if the D/A output is set to maximum. Diode laser current is temperature sensitive, and the A/D portion of the card is used to monitor the current so that power can be reduced automatically if it approaches the safe current limit. The laser power control can also be used to automatically adjust the laser power to prevent saturating the camera, though in typical operation the camera auto-shutter adjusts sufficiently that this is not needed.

The software is written in a combination of C and Perl. Perl is used as an outer scripting loop to call smaller C modules that communicate with the hardware. This software allows for easy changes to event triggers such as temperature and time-based camera operation. The camera can be read synchronously or asynchronously. For single sample images where timing is not critical, synchronous mode is used. For video rate acquisition, the camera is allowed to free-run and send images to the GigE port of the computer. Each time a frame arrives at the GigE port, it is buffered and stored to the external solid state drive. By taking advantage of the quad-core architecture, data can be acquired at the full camera capability (15 fps, 2048 x 2048 images, lossless storage) for arbitrarily long times without buffer overruns. Each arriving frame spawns a new process to write that frame to disk (with sequence and timing information preserved) and the processes are spread among the CPU cores so that they keep up with the arriving data. Video data are captured in a format and structure that allows them to be loaded directly into analysis software. Time-stamped temperature and laser data are taken and stored with each video sequence, and additionally logged regularly to diagnostic files, along with battery data.

The entire system consumes less than 15 W of power and will operate with a supply voltage of anywhere from 9 to 24 VDC input. The electronics are mounted on an aluminum plate that is coupled to two 10 cm diameter heat sinks that pass through the back of the housing and dissipate most of the heat in the external environment. The optics are mounted on a separate plate that is not thermally coupled to the electronics mounting plate.

Power is supplied by one or two 11.1 V, 99 Wh Li-Ion batteries, which are UN38.3 certified and suitable for air travel in the passenger cabin. When two batteries are used, one supplies the camera and relay board and the other the rest of the electronics. The batteries fully charge in several hours (typically overnight), and external power can be supplied from any source in the 9–24 VDC range (e.g., a car or boat battery) if the internal batteries are not available for any reason. Switching between power sources can be done in a few minutes in the field with a screwdriver.

#### Sample chambers

**Fig 4:**
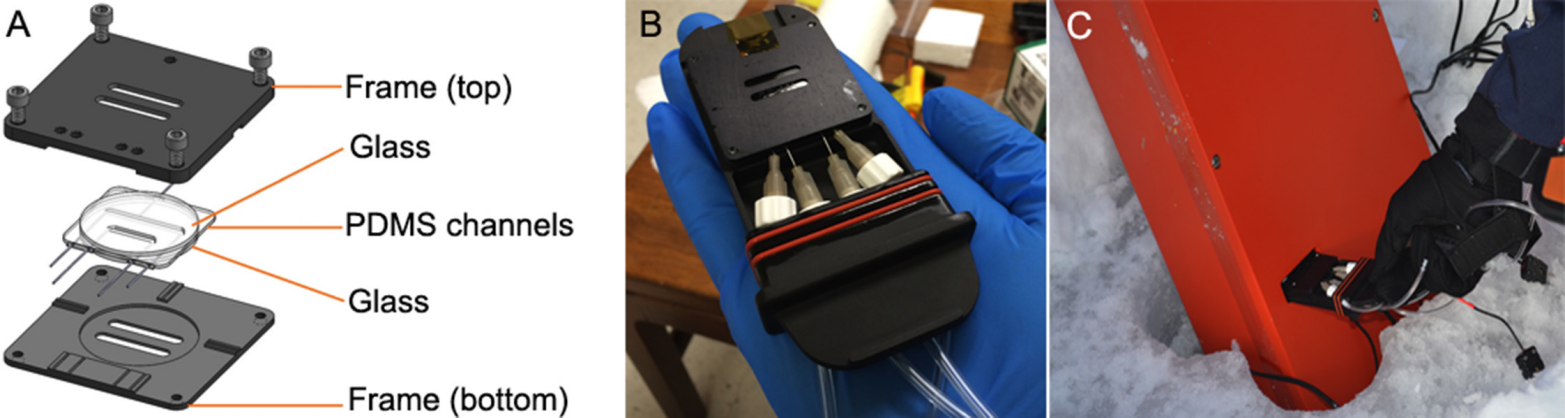
Sample chamber design. (A) Chambers had two PDMS channels, one for the reference beam and one for the object beam. (B) The chamber and plumbing fit into a cartridge with a gasket for sealing it into the instrument. Hand for scale. (C) Sample chamber being placed into the closed and sealed instrument.

The microfluidic chambers used for the results presented here were designed to fit the instrument without leaking and had two parallel channels, one for the sample and one for a fluid-only reference. The volume of each channel was 24 μL. They were designed so that light passed only through high-quality glass (**Fig 4A**). To use the chambers on site, a sample was collected and immediately delivered to the chamber with a syringe. The chamber and plumbing were held in a cartridge for leak-proof insertion into the field instrument (**Fig 4B**). Chambers were re-usable and were disassembled each day and cleaned with ethanol and water, then reassembled for subsequent use. A total of 10 chambers were made and brought to each field site.

### Instrument deployment

#### Sampling locations and methodology

**Fig 5:**
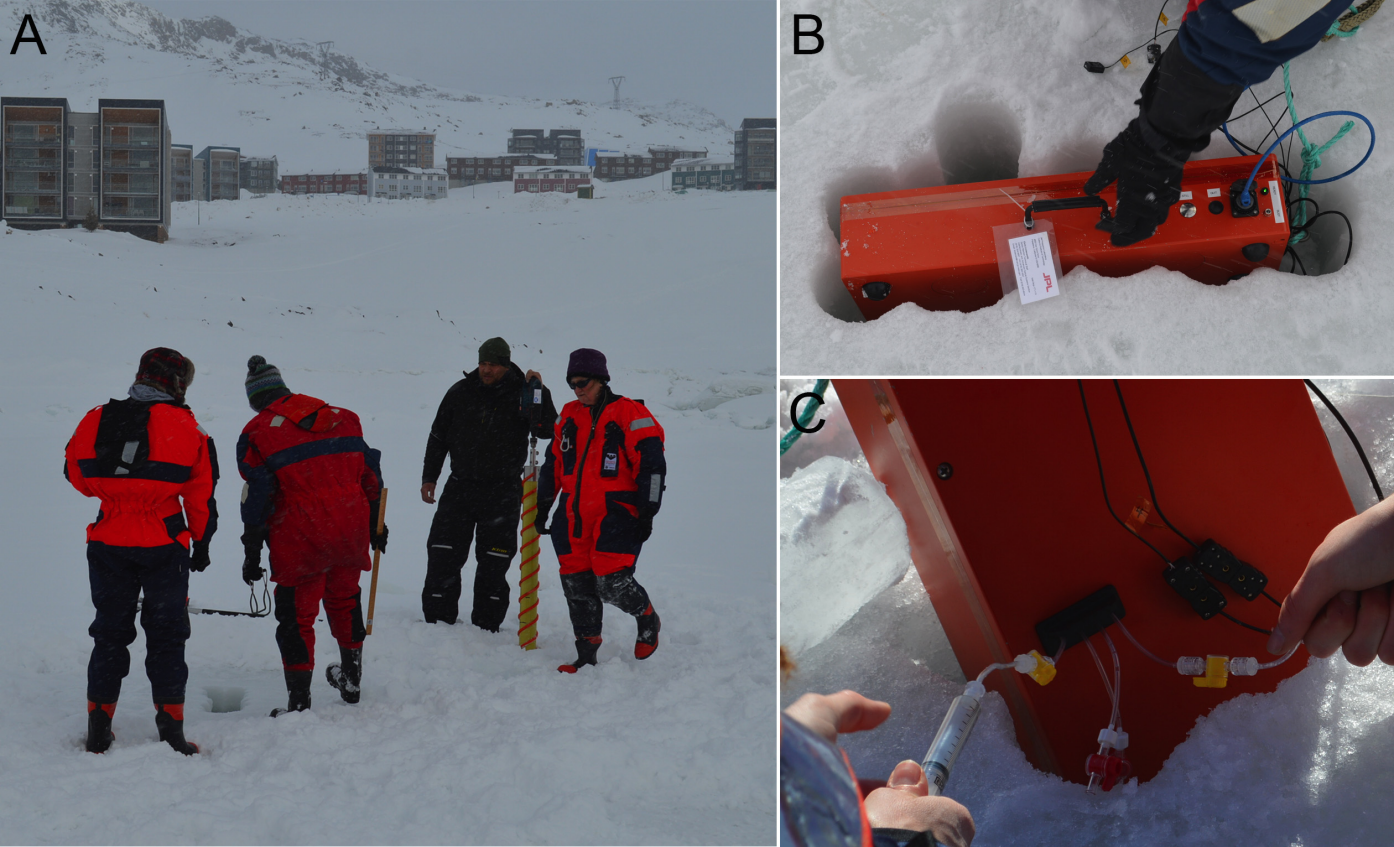
Field deployment. (A) Sackholes large enough to accommodate the instrument were drilled in sea ice with a Mark II ice corer to a depth of 20–25 cm, determined by first drilling test holes with an augur to determine ice thickness. (B) DHM in ice sackhole. (C) Sample from sackhole delivered to sample chamber via syringe. The box remains in the ice hole to keep the instrument equilibrated to *in situ* brine temperatures.

Malene Simon, director of the Greenland Climate Research Center, arranged all permissions needed for the fieldwork. The instrument was used at the end of the winter season to investigate microbial motility in the brines of sea ice, accessible from three sites near Nuuk, Greenland: Malene Bay (N 64.1707, W 51.6752); a site near the Nuuk Municipal Swimming Pool (N 64.1796, W 51.7033); and the more remote Kobbefjord (N 64.1765, W 51.5501). Air temperatures during the sampling period of 24–27 March 2015 ranged between –9°C and – 15°C; snow cover over the sea ice varied in thickness: 5–22 cm at Malene Bay, 20–25 cm at Kobbefjord, and 25–27 cm at the Municipal Pool site. Snow was cleared and sackholes were cored to a depth of 20–30 cm in ice that was 43–57 cm thick, and brine was left to drain into the sackholes for 1–2 hours (**Fig 5A**). Brine samples were collected into a sterile 60-cc syringe and sometimes concentrated in a four to one ratio over a 0.2-μm polycarbonate filter. The raw or concentrated sample was injected directly into the sample chamber of the digital holographic microscope, which had been placed in a sackhole to equilibrate the sample chamber with surrounding brine temperature (about –4°C) (**Fig 5B and 5C**). Thermometers were placed in the inlet and outlet tubing near the sample chamber, as well as in the sample chamber housing, to monitor the temperature at which microbial behavior was observed and recorded.

Additional brine was collected with ethanol-sterilized instruments and returned to the Greenland Center for Climate Research in Nuuk where it was kept on ice until processed in a temperature-controlled room (4°C) within 24 hours of collection for ancillary measurements of bacterial abundance and concentrations of chlorophyll *a* (Chl *a*) and dissolved organic carbon (DOC). Samples for bacterial abundance were fixed with 2% volume formaldehyde and kept refrigerated until returned to the laboratory at the University of Washington in Seattle, where they were vacuum-filtered onto 0.22-μm filters and stained with acridine orange and DNA-specific 4’-6’-diamidino-2-phenylindole (DAPI) (20 μg ml^–1^) according to the dual-staining technique of Schmidt *et al*. (1998) and counted using a Zeiss Universal epifluorescence microscope [41]. Samples for Chl *a* analysis were prepared and analyzed according to Arar and Collins (1997), except that particles were allowed to settle, rather than being centrifuged, prior to reading fluorescence. Samples for DOC concentration were filtered through a 0.2-μm Sterivex filter into an EPA vial and frozen until analyzed using Standard Method 5310 (American Society for Testing Materials, 1994) on a Shimadzu TOC-Vcsh carbon analyzer at the University of Washington.

#### Test conditions

At all sites, datasets were taken for raw brines, as collected, for filter-concentrated brines, and for brines with added sugar (trehalose). At Malene Bay, we also investigated new ice that had formed over open areas of water several meters from the sackhole (the previous ice cover had been broken up in a storm overnight). At both Malene Bay and Kobbefjord, cores were drilled through the ice to the seawater below, which allowed for collection of samples of the bottom layer of ice (“bottom ice”) and the seawater; these samples were also placed into the instrument for evaluation and recording.

Malene Bay brine samples collected into sterile 50 cc test tubes were returned to the laboratory and incubated overnight in cold rooms at +4°C and –4°C with or without addition of the amino acid serine, the sugar trehalose, or a complete rich medium (Difco marine broth 2216) to intentionally stimulate activity. Recordings were taken the following day. Aliquots of these same samples were fixed with paraformaldehyde or glutaraldehyde for later qualitative examination by light and electron microscopy.

Chemotactic response was analyzed in the laboratory by introduction of the chemoattractant serine in a gradient. Serine (L-Ser, > 99% purity, Sigma Aldrich) was dissolved in seawater at 0.001 M, filter-sterilized, and flowed through a chamber filled with (previously unamended) Malene Bay brine samples which had been kept at −4°C (approximate *in situ* temperature) for three days. Handling of all samples was using ethanol-sterilized or sterile pre-packaged instruments and filter-sterilized solutions. Additional chemotaxis experiments were performed by placing a crystal of L-Ser or aspartate (L-Asp, > 99% purity, Sigma Aldrich) in a sample chamber and then flowing through Malene Bay brine samples. Experiments and recordings were performed at +4°C.

### Data analysis

Hologram reconstruction into amplitude and phase images was performed using Koala (LynceeTec). Analysis of reconstructed images was performed using Fiji [42]. As a general rule, reconstruction was performed on only one z plane for illustration of motility patterns. In some cases, reconstruction was performed across the depth of the sample chamber and x, y, z, t hyperstacks were concatenated. Unless otherwise stated, images shown have been median-subtracted but have undergone no other processing. Chemotaxis analysis was performed using manual tracking with MTrackJ [43] and the Chemotaxis and Migration Tool (Ibidi).

## Results

### DHM: General

Over 140 datasets, representing > 100 Tb of reconstructed 4-D data, were collected. Data were collected over multiple test sites and conditions in a single day. A sample was introduced, recordings were taken 2–5 times for 30–60 s each, and then the sample was changed. A “snapshot” mode that acquires 3-s recordings was generally sufficient to identify probable organisms, but longer recordings enabled analysis of individual and collective swimming behavior. A single-frame mode was tested in the lab, but does not provide insight into motion of the 3-s recordings; with only a small savings in time, it was not used in the field.

Analysis of these datasets indicated that all samples contained both eukaryotes and prokaryotes. Nearly all datasets contained useful images; 4 recordings were unusable due to ice crystal formation in the sample chambers or excessive drift. The presence of ice resulted in complete image opacity, and once frozen, samples in the chambers did not thaw since the instrument was at ambient (subzero) temperatures. Because samples were delivered to an enclosed chamber with small inlet ports, there were no issues of turbulence or currents in the source liquid. Most recordings showed some degree of sample drift, which proved desirable as it allowed for visualization of a greater sample volume and for easy discrimination between particles and noise.

No problems with the instrument were encountered during travel or deployment in the field. The microscope was carried as carry-on luggage outbound, and checked in a hard-sided suitcase surrounded by clothing upon return. It was also transported by snowmobile in the field. Batteries were sufficient for the longest field day (> 8 hours). The only technical challenge in the field was occasional ice formation in the plumbing lines leading to the sample chamber, which could be remedied by manual warming. In the laboratory, icing occurred on the optics when the instrument was placed in a –4°C cold room and the door was opened and closed to change samples, introducing warm humid air to the room. This problem could be remedied by equilibrating the instrument in the room and changing samples without opening and closing the door. Samples were always stored at the temperature of the room.

### DHM: Eukaryotes

**Fig 6:**
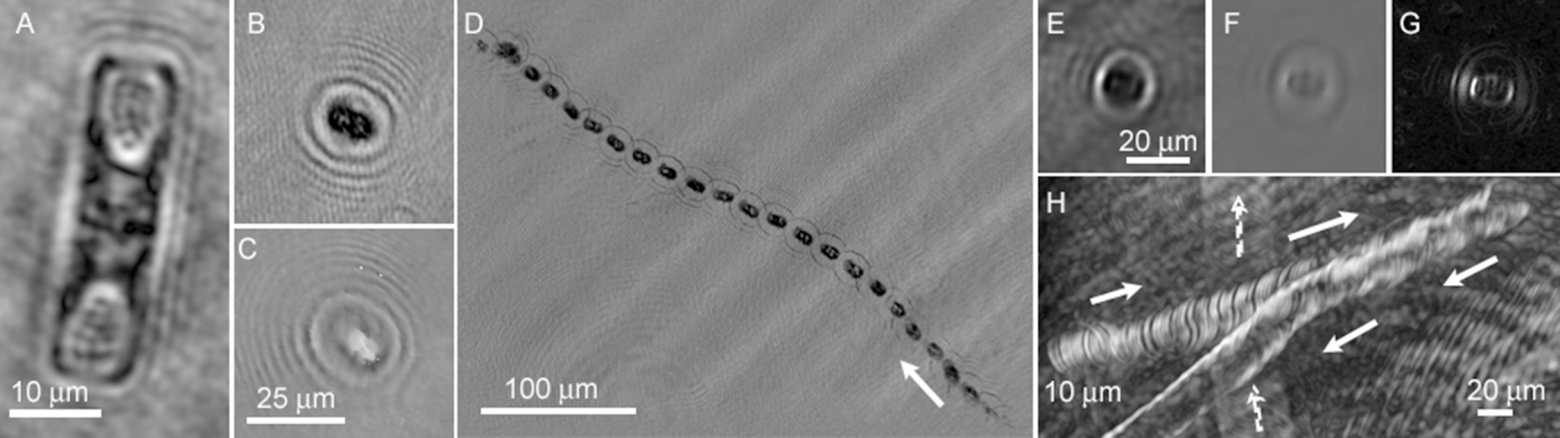
Examples of eukaryotic cells and trajectories observed in sackhole brines of Malene Bay. (A) Amplitude image of non-motile diatom. Note the clear demarcation of cell walls and organelles. (B, C) Rapidly swimming organism in amplitude (B) and phase (C) (the two images do not represent the same time point, but instead the best focus at the given z-plane, which differed in intensity vs. phase). (D) Minimum intensity amplitude projection giving the trajectory of the organism in (B,C) over 7 s. The arrow indicates the direction of motion. (E-G) More slowly swimming, typical organism seen in amplitude (E), phase (F), and the derivative of phase (G). (H) Trajectory of organism in (E-G) over 110 s (arrows). The cell reversed direction near the edge of the field. Note the trajectory of another, out-of-focus organism in the background (dashed arrows).

Analysis of the > 100 useful datasets showed eukaryotic motility in all samples. More eukaryotic cells were seen in new ice and bottom ice than in sackhole brine, but cells were always immediately apparent in all of the tested sample types. Thus, all tested brines, whether from sea-ice sackholes, freezing surface seawater or bottom ice and seawater, were “positive” for life upon immediate inspection in the field. A significant fraction of the eukaryotes were non-motile, most notably the diatoms, which do not swim. The cell structure of the diatoms, however, provided an excellent biosignature even in the absence of motility. A diatom from Malene Bay brine is shown in **Fig 6A**. Motile eukaryotes were seen ranging in size from 15 to 50 μm. The presence of chlorophyll could be observed as dark areas in amplitude and phase, as we have reported previously [40]; these dark areas are the result of strong absorbance of chlorophyll at 405 nm. Most of the motile eukaryotes had evidence of chlorophyll. One of the larger, more rapidly swimming organisms observed is shown in amplitude in **Fig 6B** and phase in **Fig 6C**. Its trajectory over 7 s of video is seen in **Fig 6D** (also see **S1 Video**); its speed was approximately 50 μm s^–1^ under *in situ* conditions in Malene Bay brine. This speed represented the upper limit of speeds seen at these subzero temperatures. Typical motile cells were smaller, photosynthetic (given dark areas), and spherical. **Fig 6E, 6F and 6G** show amplitude, phase, and phase derivative images of a typical cell from Malene Bay new ice, and **Fig 6H** shows its trajectory over 110 s of recording (also see **S2 Video**), with a speed of ~ 5 μm s^–1^.

There were no false positive results for motility; meaningful motion was clearly distinguishable from Brownian motion and sample drift in all cases. The organism density was low in most samples (~10^4^–10^5^ cells mL^–1^), so concentration (by filtration) was helpful to obtain more organisms per field. Concentration was not necessary to observe motility.

### DHM: Prokaryotes

**Fig 7:**
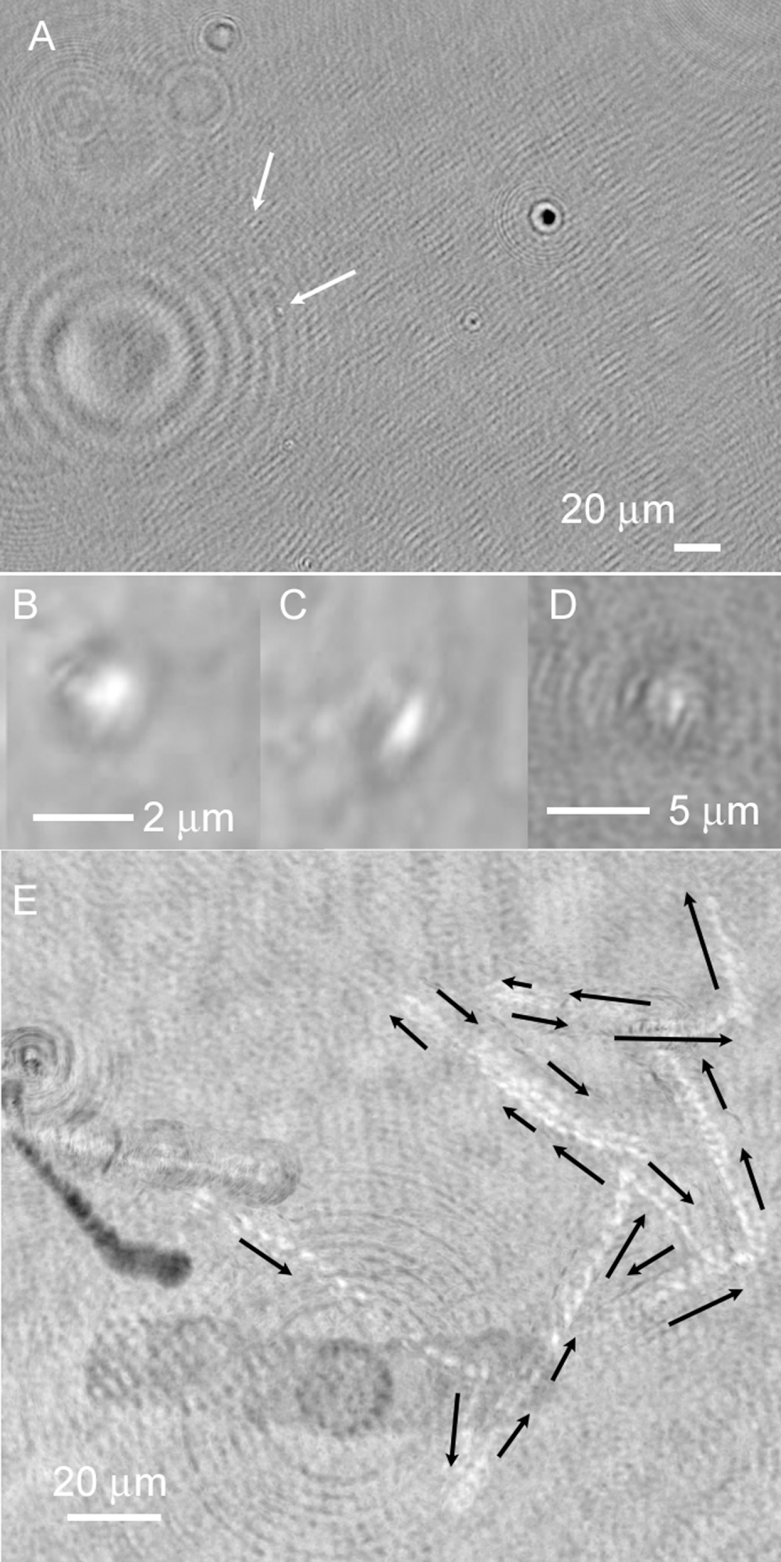
Examples of prokaryotes and trajectories observed from Malene Bay seawater. (A) Appearance of a nearly full-screen image containing objects suggestive of prokaryotes (arrows). (B) Zoomed-in appearance of a cell just out of the focal plane. (C) A cell at best focus. (D) Appearance of a prokaryote in a maximum intensity z-projection through 10 z planes (24 μm). The apparent size and contrast are increased. (E) Zig-zag motility of a prokaryote, observed as a maximum intensity projection through 60 s of time on a single z plane.

Under ordinary conditions of imaging and reconstruction, prokaryotes could not be distinguished definitively from debris if they were not motile. Bacteria also resembled laser speckle noise, but Brownian motion or flow in the sample chamber was sufficient to distinguish particles from noise as the particles moved passively across the background (see **S3 Video**). Still images were suggestive but not conclusive of prokaryotic cells (**Fig 7A**). Unlike the photosynthetic eukaryotes, the prokaryotic cells did not show dark areas with 405 nm illumination; instead, their greatest contrast was seen in amplitude images as brighter than background (**Fig 7B** and **7C**). Projection of several z-planes increased the effective size of the cells and facilitated their visualization (**Fig 7D**). When it occurred, bacterial swimming was clearly distinguishable from Brownian motion and sample drift due to greater speed, swimming “upstream,” and/or reversals of direction (**Fig 7E**) (see also **S4 Video**).

**Fig 8:**
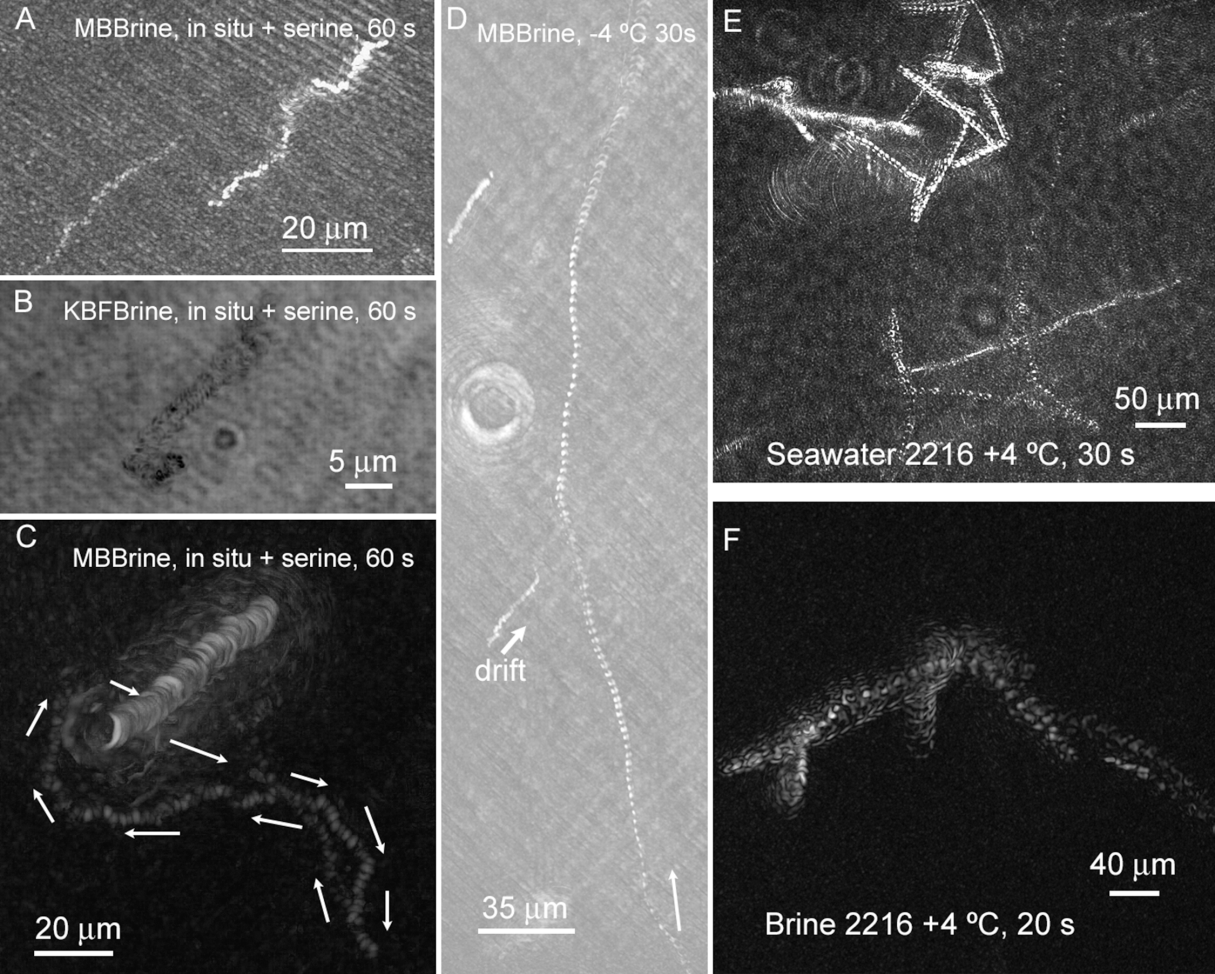
Examples of trajectories of prokaryotic motility seen in Nuuk samples. Images are maximum- or minimum-intensity projections across the length of time indicated in each image. (A) Malene Bay brine measured *in situ* with serine added to the sample chamber. (B) Kobbefjord brine measured *in situ* with serine added to the sample chamber. The ambient air temperature was –12°C. Note the non-motile organism for reference. (C) Another sample of Malene Bay brine measured *in situ* with serine added. (D) Malene Bay brine sample returned to the lab and stored at –4°C with measurement performed at –4°C. (E) Seawater sample warmed overnight to +4°C with the addition of half strength 2216 Marine Broth medium. (F) Brine sample warmed overnight to +4°C with the addition of half strength 2216 Marine Broth medium.

The majority of prokaryotes, however, were non-motile under *in situ* conditions in the images of sea ice brines reported here. Over 50 videos were taken in which the characteristic swimming patterns of marine bacteria were conspicuously absent. No prokaryotic swimming was observed at any of the sackhole brines under *in situ* temperatures without the addition of nutrients, though motility could be seen in samples extracted from the bottom of the ice core (which was regularly flushed with seawater) and from seawater. The addition of serine led to observable motility in both Malene Bay and Kobbefjord brines *in situ*. The swimming speeds were usually very slow (~1 μm s^–1^), characteristic of gliding or twitching motility rather than flagellated swimming (**Fig 8A** and **8B**). However, occasionally swimming at ~5 μm s^–1^ was seen. Motile prokaryotes could be observed to “track” larger organisms (**Fig 8C**) (see also **S5 Video**).

When samples were returned to the laboratory, changes in temperature and nutrient concentration were used to examine their influence on presence and speed of prokaryotic swimming. A dramatic increase in swimming speed was seen when samples were warmed to +4°C, with prokaryotic speeds of up to 50 μm s^–1^ apparent. When samples were stored at −4°C, one hour incubation at +4°C was sufficient to increase swimming speeds. In some cases, very rapid swimming was seen as low as −4°C (**Fig 8D**) (also see **S6 Video**). However, in the absence of supplementation, the number of motile prokaryotes remained low. The use of complete marine broth medium (half-strength Difco Marine Broth 2216), combined with incubation overnight at +4°C, resulted in large numbers of highly motile prokaryotes demonstrating classic zig-zag motility in seawater samples (**Fig 8E**) (also see **S7 Video**). In brines, motility percentages remained low even when full medium supplementation was used. When motility was seen, it was rapid and characteristic (**Fig 8F**) (also see **S8 Video**), but no significant increase was seen in rich medium at 4°C vs. warming alone.

### Chemotaxis

**Fig 9:**
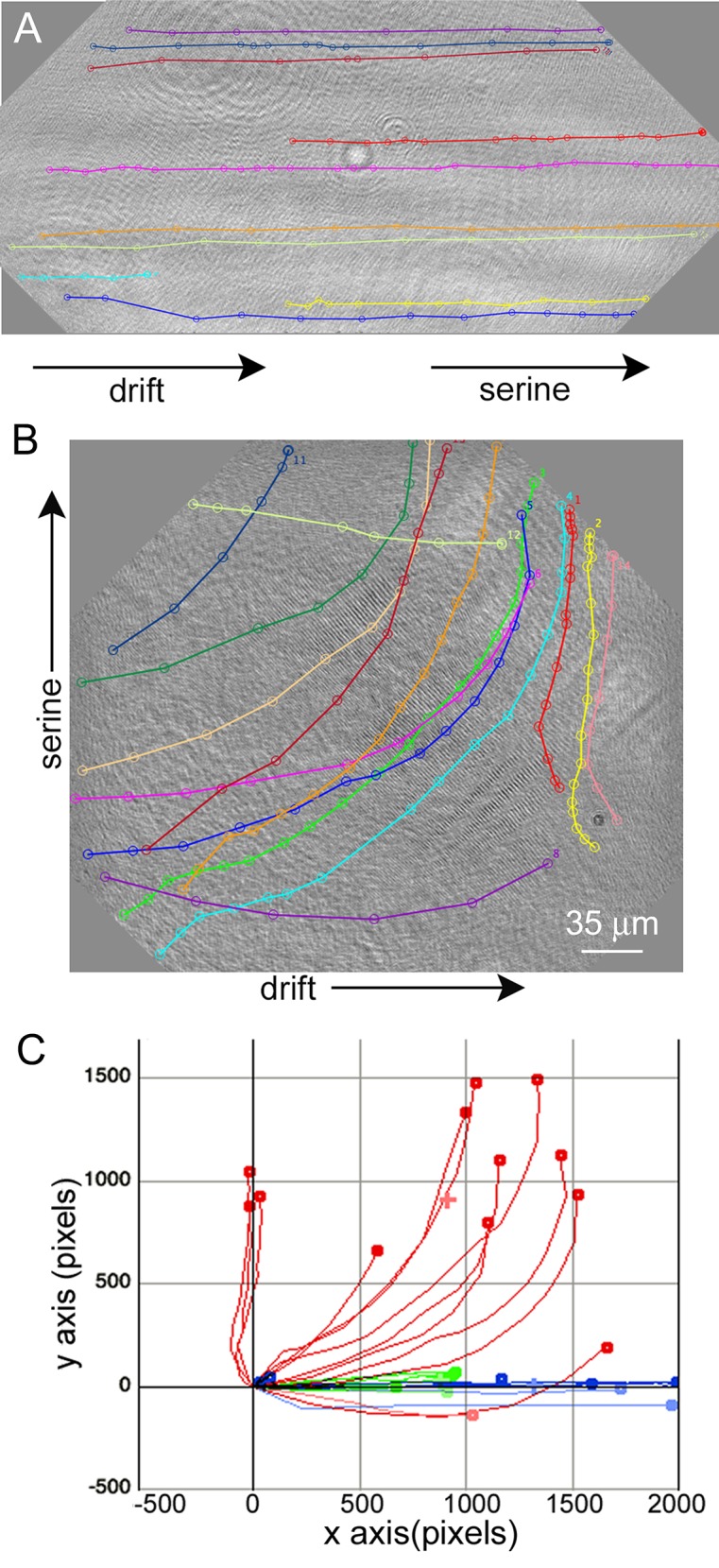
Appearance of tracks of identified cells (prokaryotes and eukaryotes) in the presence of unequal serine concentrations. The images are rotated 45° to reflect the orientation of the chamber in the microscope. (A) Serine introduced at the right edge of the sample chamber. (B) Serine introduced in the center top of the sample chamber. (C) Chemotaxis plot showing controls with drift and no serine gradient (green), cells from Panel A in blue, and cells from Panel B in red. Calculated x and y velocities of the cells are given in **Table 1**.

The generation of a serine gradient within the sample chamber led to directed motility in the direction of the high end of the concentration gradient (**Fig 9**) (**S9 Video**). When serine was introduced at the edge of the chamber and thus aligned with chamber drift, overall speeds increased without change in direction (**Fig 9A** and **9C****; Table 1).** Taxis could be quantified most easily when the serine was introduced perpendicular to the direction of chamber drift (**Fig 9B** and **9C****; Table 1**).

**Table 1:** Total velocity, longitudinal (x) and transverse (y) velocities, and range of velocities seen for the samples pictured in Fig 9, as well as a control without serine.

Condition (# of cells)	Mean ± SD velocity (pixel s^–1^)	V_x_ (pixel s^–1^)	V_y_ (pixel s^–1^)	Velocity range (pixel s^–1^)
Control (10)	17 ± 6	16 ± 6	–1 ± 1	12–26
Serine at edge (13)	240 ± 90	230 ± 90	–17 ± 50	85–357
Serine at top center (14)	100 ± 20	60 ± 40	–70 ± 30	66–138

Note greatly increased overall velocities in the presence of serine and directionality conferred by adding serine perpendicular to the direction of flow.

### Light microscopy and community analysis

**Fig 10:**
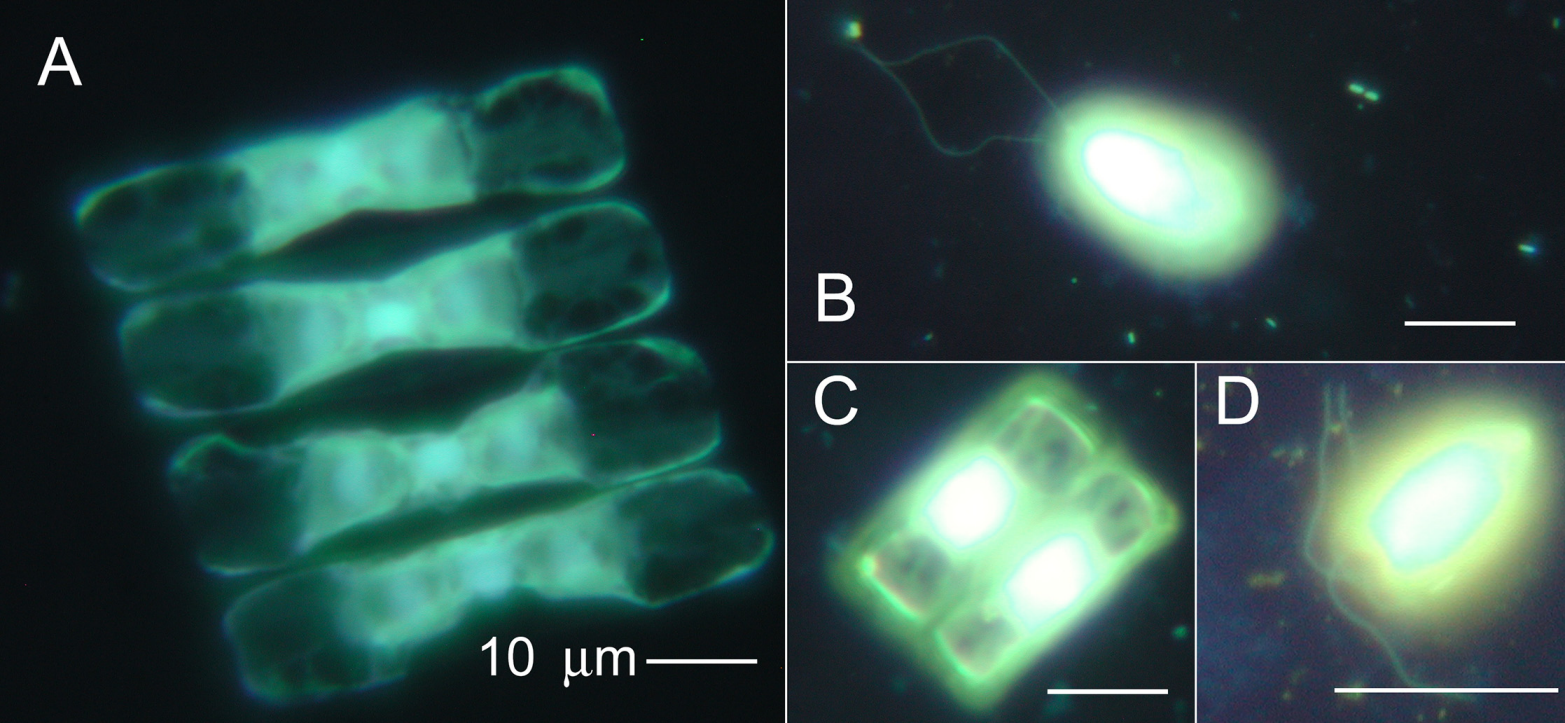
Images by epifluorescence microscopy of brine samples. Organisms are stained with acridine orange and DAPI according to a dual-staining technique [41] and showed an assemblage of diatoms (A, C) and flagellates (B, D) from Malene Bay (A, B) and Kobbefjord (C, D). All scale bars are 10 μm. From Malene Bay: (A) A diatom, probably *Navicula vanhoeffenii* and (B) a flagellate, possibly *Chlamydomonas* or *Telonemia*; from Kobbefjord: (C) a diatom, probably *Fragilariopsis oceanica*, and (D) a flagellate, *Pyraminmonas sp*. *(*http://westerndiatoms.colorado.edu/) [46] [47].

Examination of samples by light microscopy confirmed the presence and appearance of the organisms seen by DHM: non-motile diatoms containing chlorophyll, motile flagellated eukaryotes, and prokaryotes 1–2 μm in length (**Fig 10**). Bacterial abundance, determined by epifluorescence microscopy, was typical for late-winter sea ice [8], ranging between 9.44 x 10^4^ and 4.34 x 10^5^ cells ml^–1^ (mean = 3.03 [± 1.25] x 10^5^ ml^–1^, n = 19). Concentrations of Chl *a* were variable but low (mean = 3.20 [± 2.02] μg L^–1^, n = 14), as observed in prior studies of sea ice from these sites [44] [45]. DOC concentrations varied widely (mean = 36.1 [± 27.7] mg L^–1^, n = 5), as observed in other sea-ice studies [33] [34]. Overall the brines examined in this study represented a low to moderately nutrient-rich environment.

## Discussion

Microbial life on Earth is identified and classified with the aid of light (brightfield, phase-contrast, epifluorescence) or electron microscopy, usually in conjunction with dyes or stains such as the Gram stain, 4',6'-diamidino-2-phenylindole (DAPI), or other molecules that target specific cell structures. These techniques permit the distinction between cells and nonliving particles such as mineral grains, but they are difficult to implement in space for several reasons. The instruments are large and fragile, the techniques require washing, and planetary protection requirements will likely prohibit the use of dyes and stains on Planetary Protection Class IVb missions (those intending to investigate extant Martian life) and Class IVc missions (those visiting Martian Special Regions which are most likely to contain extant life or to permit replication of imported Earth life). Thus, the development of space-worthy, label-free microscopic techniques is necessary for any mission intending to search for extant microorganisms on Mars or elsewhere.

We do not yet have a full picture of the usefulness of motility as a biosignature on Earth, because *in situ* microscopy has rarely been performed in any environment, including the open ocean and ice habitats. In order to fully appreciate and use this compelling, non-Earth-centric biosignature, we must first explore the wide variety of analog environments on Earth where microbial motility may or may not be present. With the recent confirmations of accessible water ice on Mars and likely seasonal liquid water streams, along with increased interest in the ice and oceans of Europa and Enceladus, exploring terrestrial analogs is imperative.

Most marine organisms are capable of motility, though only a portion of them may be swimming *in situ* at any given time [48]. Overnight incubation in rich culture media will lead to a majority of motile organisms [49]. Our results are consistent with this expectation: e.g., the seawater collected from under the ice showed a large number of highly motile prokaryotes after overnight incubation in 2216 marine broth at +4°C. This work is the first report of immediately examining brines, collected directly from the ice, for motility. Only a small number of motile prokaryotes were seen in these samples, however, and only after addition of nutrients, warming to +4°C, or both. Even in these cases, only a few highly motile organisms showing the classic zig-zag swimming characteristic of marine bacteria were seen. These results do not diminish the value of motility as a biosignature, but do mean that a sufficient number of samples must be examined and recordings must be continued long enough to observe the motile organisms. The possibility of contamination must also be considered. The medium used for amendment here (half-strength marine broth) and the low temperatures used for incubation argue against growth of human-derived contaminants. It is also unlikely that the motility observed reflects enrichment of a few motile organisms because of the timescales involved. The most rapid doubling time of the most cold-adapted of marine psychrophilic bacteria from sea ice at 4°C in marine broth is about 12 hours; it is >> 4 days at –4°C and seawater salinity [50, 51], so no detectable growth would have occurred at –4°C for even the longest period (3 days) that we kept brines stored, and especially not in samples that were not previously amended with any of the substrate solutions. For life detection experiments, strict sterility of all surfaces and solutions that come in contact with the targeted sample will be of the utmost importance.

Permissive parameters for observing motility in such extreme environments will be constrained by further experiments in extreme environments, including other types of sea ice, permafrost, and glacier ice. Sea ice has the notable property of allowing for collection of interstitial brines without melting the surrounding ice. Other types of ice, such as glacier ice, will require melting before imaging. As melting will dilute the organisms present, detection may be more difficult unless a significant concentration step is also included. It will also be important to incorporate heating into the sample chamber design so that samples may be warmed during observations on site.

Conventional label-free focused light microscopy suffers from several drawbacks as a life detection technique. Its throughput is usually low, with limits of detection at ~10^5^ organisms mL^–1^ under typical conditions. More seriously, it cannot inconclusively distinguish prokaryotes from minerals or debris using morphology alone. DHM resolves these issues by recording, in a compressed way, the entire contents of a relatively large sample volume with each hologram in both intensity and phase, allowing detection of substantially transparent objects due to small index of refraction differences from the medium, reducing or eliminating the need for labeling or special sample processing. Because the DHM can image the entire sample volume at relatively high speed without special processing, it can allow for high throughput by using a small pump to move sample into and out of the sample chamber quickly, significantly lowering the limit of detection. Background currents can be eliminated by the addition of microvalves on the inlet and outlet if necessary, though in a space system the risk of valve failure may outweigh the small benefit of suppression of background flow. DHM represents an alternative to the Imaging FlowCytobot, a flow cytometer for marine applications that both counts and images eukaryotic cells [52, 53]. The FlowCytobot images cells rapidly one at a time, thereby eliminating problems of current and throughput. However, it is so far restricted to organisms > 10 μm in size. Another advantage of DHM is that it can capture many aspects of the native environment, so that processes such as predation may be observed on the micrometer scale [54].

Additional features may be added to holographic microscopy in order to increase its value as a label-free life detection technique. The use of 405 nm clearly indicates chlorophyll on Earth, but for extraterrestrial use, multi-wavelength illumination could provide visible spectra of any cell-like objects and thus help detect biosignature pigments such as carotenoids and rhodopsins. Although the DHM used for these studies is monochromatic, the system described above can be extended to support multispectral observations, either with a tunable light source, or multiple monochromatic light sources. The sample chamber design described here also supports the addition of various stimuli to investigate taxis in microorganisms, which can be very strong indicators of extant life. Phototaxis, magnetotaxis, chemotaxis, and thermotaxis can be added with small modifications to the system. An additional, though somewhat extreme, test of extant motile life in a cryoenvironment is to simply close off the sample in the chamber and heat it sufficiently to kill any present organisms and compare activity before and after. If the sample comes from an external reservoir, the chamber can then be flushed with a new sample containing new microorganisms to confirm the observations. In all possible cases, the sample can be subsequently fed into a chemical analysis system to determine the presence of chemical biomarkers, providing independent data to confirm or exclude the possibility of the discovery of extant life.

## Summary and Conclusion

The role of bacterial motility in icy environments remains almost wholly unexplored, despite such environments serving as important analogs for astrobiology-related exploration. The yield of this work was both microbiological (indicating that eukaryotic motility in trapped brines occurs, and prokaryotic motility occurs with stimulation) and astrobiological (supporting the use of DHM as a life-detection technique with essentially no false positives). Further characterization of *in situ* motility in other environments is needed to know whether most or all Earth environments demonstrate the definitive motility seen in the sea-ice brines of this study. Expanding this type of evaluation will have important implications for detection of life on the moons of outer planets.

The use of DHM may provide significant insight into microbial motility that, until now, has not been feasible to observe *in situ* in extreme environments. Motility has implications to key processes such as the origin and early evolution of life on Earth (and elsewhere) [33]; nutrient cycling in the oceans [48] [55]; biofouling; and microbial pathogenesis. Current experiments require removal of samples from their natural settings, with corresponding disruption to *in situ* conditions and a delay in time before imaging can be performed. Traditional focused-light microscopy also suffers from limited depth of field, and limited field of view, making it difficult or impossible to track significant numbers of organisms in three dimensions, especially in highly dilute samples. The instrument reported here is inexpensive to build, robust, and designed to be carried within limited aircraft luggage space, and thus represents a practical approach to measurements of *in situ* motility in the most remote locations.

## Supporting Information

S1 Video Rapid eukaryotic motility in Malene Bay brine. (MOV)

S2 Video Slower eukaryotic motility in Malene Bay brine. Note the sample drift almost perpendicular to the organism’s swimming.
(AVI)

S3 Video Brownian motion. Motion of particles against the background may be used to distinguish them from optical noise.
(AVI)

S4 Video Prokaryotic motility. This seawater sample illustrates the classic zig-zag motility of marine bacteria. The images are projections through 10 z planes.
(AVI)

S5 Video Motile prokaryote in serine-fed brine. (AVI)

S6 Video Rapid prokaryotic swimming at –4°C. This sample of Malene Bay brine was transported from field to laboratory and stored in a –4°C cold room with no other treatments.
(AVI)

S7 Video Motility in seawater warmed to +4°C overnight with addition of 2216 marine broth. Note the large number of motile prokaryotes.
(AVI)

S8 Video Motility in brine warmed to +4°C overnight with addition of 2216 marine broth (AVI)

S9 Video Motility in a brine sample exposed to a gradient of serine (gradient runs bottom-to-top). (AVI)
